# Evaluation of the Oral Microcirculation in Patients Undergoing Anti COVID-19 Vaccination: A Preliminary Study

**DOI:** 10.3390/vaccines10111978

**Published:** 2022-11-21

**Authors:** Adriana Acquaro, Giorgia Brusca, Sofia Casella, Enzo Maria Cumbo, Antonio Della Valle, Mohmed Isaqali Karobari, Giuseppe Marino, Anand Marya, Pietro Messina, Giuseppe Alessandro Scardina, Domenico Tegolo, Antonino Tocco, Cesare Valenti

**Affiliations:** 1 Department of Surgical Oncological and Stomatological Disciplines, University of Palermo, 90127 Palermo, Italy; 2 Department of Conservative Dentistry & Endodontics, Saveetha Dental College & Hospitals, Saveetha Institute of Medical and Technical Sciences University, Chennai 600077, Tamil Nadu, India; 3 Department of Mathematics and Informatics, University of Palermo, 90123 Palermo, Italy; 4 Department of Orthodontics, University of Puthisastra, Phnom Penh 12211, Cambodia

**Keywords:** COVID 19 vaccine, SARS-CoV-2 infection, capillaroscopy, microcirculation

## Abstract

Videocapillaroscopy allows the study of both the morphological and architectural structure of the microcirculation and its hemodynamic conditions; these parameters are directly involved in autoimmune and/or inflammatory pathologies. The purpose of this research, based on capillaroscopy, is to establish whether a patient who receives an anti-COVID 19 vaccine has any changes in their oral microcirculation. A complete capillaroscopic mapping of the oral cavity of the subjects examined was made; the investigated mucosa sites were the following: cheek, labial, chewing-gingival and back of the tongue. This study showed an increase in capillary density from the comparison between the mean labial capillary density of vaccinated patients and the reference mean capillary density value of the literature. The increase in capillary density is a sign that can be attributed to an increase in angiogenic activity. The EMA, GACVS and MHRA have reviewed the risk of thrombosis after vaccination, agreeing that the benefits outweigh the risks.

## 1. Introduction

**Figure 1:**
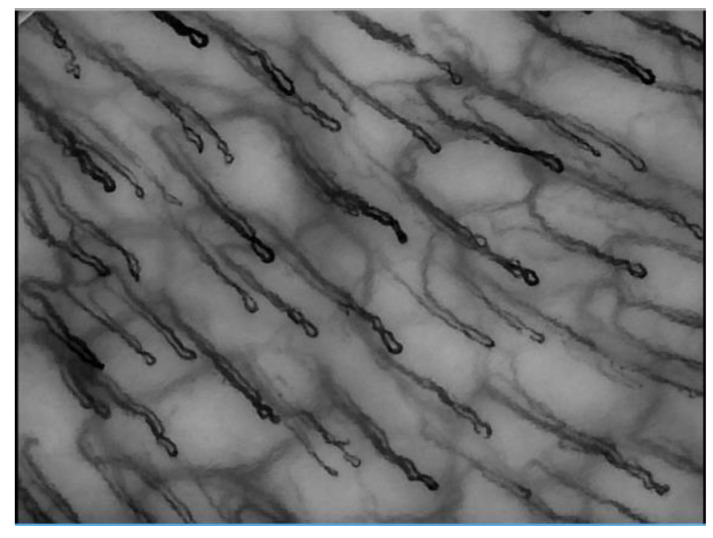
Sample still frame of a typical videocapillaroscopic acquisition (150× magnification).

The morphological study of microcirculation is fundamental because the micro-vascular bed is directly involved in both autoimmune and inflammatory pathologies. Videocapillaroscopy has been widely used for studies on the microcirculation of the oral cavity in patients with inflammatory and/or autoimmune diseases. The alterations of a microcirculation, in a given district, are often superimposable to those of other districts and, therefore, any alterations observed in correspondence of the mouth can be an expression of pathologies not exclusively pertaining to the oral cavity, but also of systemic relevance and vice versa. [1,2,3,4]. In addition, the videocapillaroscopic examination is a non-invasive, low-cost, simple to perform test and is absolutely suitable for screening, diagnosis and monitoring of various diseases [5] Figure 1. Angiogenesis plays a key role in the pathogenesis of inflammatory diseases, providing not only the proliferation of new vessels that allow better oxygenation and a greater supply of metabolites to the proliferating tissue, but also considerably increasing the complex system of feedback and turnover of the cells involved in the inflammatory process [6,7]. This feedback has been documented in other studies which have revealed the presence, in isolated endothelial cells, of the pre-eminent factor in the angiogenic phenomenon: vascular endothelial growth factor (VEGF). SARS-CoV-2 infection, also known as COVID-19, is a viral disease, which can be associated with cardiovascular complications such as venous and arterial thrombosis [8,9,10,11,12,13]. The viral spike protein, in fact, can promote the release of pro-thrombotic and pro-inflammatory mediators; vaccines, which encode this protein, are the main way to prevent COVID-19. However, some unexpected thrombotic events at particular sites, such as the cerebral or splanchnic venous circulation, have been observed in subjects, especially fertile women, who have received adenovirus-based vaccines. This clinical entity has been recognized as a new syndrome called vaccine-associated immune thrombocytopenia, possibly caused by cross-reacting antibodies against platelet activating factor 4 (PF4). The COVID-19 pandemic disease has stimulated the development, in an extremely short time, of effective vaccines directed against the SARS-CoV-2 virus, based on different technologies. Four COVID-19 vaccines were developed and approved by the European Medicines Agency (EMA) from December 2020 to March 2021 [14].

Two vaccines are based on mRNA encapsulated in lipid nanoparticles-BNT162B2 (Pfizer/BioNTech) and mRNA-1273 (Moderna), which code for the viral spike protein (S). Two others—ChAdOx1 nCov-19 (AstraZeneca) and Ad26.COV.2 (Johnson & Johnson/Janssen)—consist of a recombinant adenoviral vector and encode SARS-CoV-2 glycoprotein S. AstraZeneca/Oxford uses a modified chimpanzee adenovirus to contain the gene for the production of the spike glycoprotein (S). Johnson & Johnson uses the modified human adenovirus vector serotype 26, which encodes the complete sequence of S [15].

The global vaccination campaign has been accompanied by new vaccine-related complications [16,17,18,19,20,21]. The most serious of these is a new disease, described as vaccine-induced immune thrombocytopenia and thrombosis (VITT). VITT is rare, with an incidence of 14.9 cases per million after the first dose or 1.8 cases per million after the second dose [22]. Several cases have been reported of thrombotic events in atypical locations associated with thrombocytopenia with severe bleeding and sometimes signs of disseminated intravascular coagulation, in subjects receiving SARS-CoV-2 vaccination with Vaxzevria viral vector vaccines (ChAdOx1 nCov-19 Astrazeneca) and with COVID-19 Vaccine Janssen (Ad.26.COV2.S Johnson & Johnson). Thrombosis associated with the use of the ChAdOx1 nCoV-19 vaccine have peculiar clinical-laboratory characteristics, for example, a peculiarity of these thrombotic pictures was the presence of an increase in the levels of D-dimer associated with a reduction in those of fibrinogen, suggesting the possibility of the simultaneous presence of a consumption coagulopathy. These events were observed almost exclusively within three weeks from vaccination in healthy subjects younger than 60 years, predominantly women [23,24]. Based on the data available in the literature, it is estimated that one case of thrombosis occurs in atypical sites with thrombocytopenia on 100,000 vaccinated subjects. For the Vaxzevria vaccine, from EudraVigilance source, as of 4 April 2021, 169 cases of cerebral venous sinus thrombosis (TSVC) and 53 cases of splanchnic vein thrombosis, often associated with thrombocytopenia, have been reported, out of a total of about 34 million doses of Vaxzevria vaccine administered in the European Economic Area and the UK, equivalent to 6.5 cases per million subjects who received at least one dose [25]. A second vaccine that has been associated with thrombosis is the Johnson & Johnson Janssen vaccine, for which it has been hypothesized that viral vectors could play an important role [26]. Very rare cases of thrombosis have been related to mRNA vaccines [27]. In general, venous thromboembolic events occurring in subjects vaccinated with Vaxzevria and with the Janssen vaccine were actually no more frequent than those expected in the unvaccinated population.

An important pathophysiological mechanism has been hypothesized by the Greifswald Working Group, led by Andreas Greinacher [28]. This working group, describing the clinical-laboratory characteristics of the cases they observed after administration of the AstraZeneca vaccine, found similarities between this observed serious adverse effect (VITT) and heparin-induced thrombocytopenia (HIT).

Vaccination could induce the formation of antibodies against platelet antigens as part of the inflammatory reaction and immune stimulation; the adenoviral epitopes used in vaccines also have a strong affinity for PF4 and can cause massive platelet activation, increased tissue factor expression, and subsequent thrombin generation regardless of the presence of heparin [29]. The delay in the production of these autoantibodies would explain the appearance of adverse reactions 4–14 days after vaccination. The main similarity observed between AstraZeneca vaccine thrombosis, in particular, and HIT is represented by the coexistence of a thrombotic picture and an associated thrombocytopenia. However, the patients who presented this particular vaccine complication, unlike the classic HIT, had not had a previous exposure to heparin.

Another analogy is represented by the identification, carried out by means of an ELISA test, of a high titer of antibodies directed against a complex formed by the platelet protein PF4 with a polyanionic substance. This type of antibody has been identified in all described cases of thrombosis secondary to the anti-COVID-19 vaccine and would have a pathogenetic significance. The binding of the antibody with the PF4-polyanionic molecule complex, in fact, would be able to determine an intense platelet activation which, in turn, would be the cause of the thrombotic events typical of this clinical syndrome.

It has been hypothesized that even an accentuated immune-type response (mechanism that mimics the effect of active COVID-19) could represent a thrombotic trigger [30]; a disproportionate inflammation can increase endothelial adhesion, activate neutrophils (which produce NET) and monocytes, promote the release of tissue factor and thrombin (key enzyme in coagulation). Furthermore, the spike protein (whose synthesis is induced by the vaccine) or molecular components of the adenoviral vector could be among the possible causative factors that activate the complement system and can induce a cellular and humoral immune cascade favoring thrombosis [31].

According to a further hypothesis, adenoviral vaccines can infect permissive cells such as epithelial, endothelial and fibroblasts, which can secrete large amounts of S glycoproteins, leading to an activation of immunity against these cells.

MRNA vaccines, on the other hand, could directly involve platelets and megakaryocytes causing the intracellular synthesis of S proteins, which would cause an autoimmune response against these elements, causing reticulo-endothelial phagocytosis and direct lysis of CD8 T cells. Cellular debris can release a large amount of whole or fragmented S proteins into the blood and can be formed a large volume of immune complexes; all of this can culminate in thrombosis [32]. 

Finally, it is known that SARS-CoV-2 uses the angiotensin 2 converting enzyme (ACE2) to invade target cells. Vaccines have the potential to interact with ACE2, promoting its internalization and degradation (phenomenon also observed in platelets). The loss of ACE2 receptor activity from the outer side of the cell membrane leads to a lower generation of angiotensin II, which increases the thrombotic risk [33,34]. 

Rare cases of systemic capillary leak syndrome (SCLS), also known as Clarkson’s disease, have been reported following vaccination with the SARS-CoV-2 adenoviral vector or mRNA vaccine. Some cases have occurred in patients with a prior history of SCLS [35]. The etiology of this syndrome is unknown and, most commonly, develops in adults between the ages of 50 and 70; it occurs rarely in the general population. Since it was first characterized in 1960, less than 500 cases have been described in the medical literature (National Organization for Rare Disorders). The current prevalence of SCLS is estimated to be less than 250 cases worldwide [36]. Capillary leak syndrome, although exceptional, was added by the EMA as a possible adverse effect following the administration of Moderna and Pfizer/BioNTech’s Spikevax vaccine; this decision was made after evaluating all available data and all cases of the syndrome reported in the Eudravigilance database following vaccine administration [37]. Out of 55 reports of SCLS, a total of 11 adverse reactions were detected with Spikevax over approximately 559 million doses administered and 44 with Comirnaty over 2 billion injections. In addition, very rare cases of SCLS have also been reported following vaccination with Vaxzevria/Covid-19 vaccine Astrazeneca, with an estimated reporting rate of one case in more than 5 million doses administered.

Other reports have come following vaccination with another anti-Covid vaccine: Janssen, of which three cases of capillary leak syndrome have been reported (occurred within 2 days of vaccination).

Considering the potential vascular risk in patients undergoing anti-Covid-19 vaccination, the present study tried to verify, through the use of the videocapillaroscope on the mucous membranes of the oral cavity, the presence or absence of morphological and morphometric alterations in the capillary bed of the microcirculation of the oral cavity, (patients were vaccinated with Pfizer and with Moderna). This research compared the parametric data obtained from a software developed at the University of Palermo (DMI; Department of Mathematics and Computer Science) which is able to extrapolate various information from the videocapillaroscopic examinations (Minutes n.6/2012 Ethics Committee).

The purpose of this study, based on capillaroscopy, is to establish whether the patient who has had an anti-Covid 19 vaccine, also in relation to the type of vaccine itself, has any changes in oral microcirculation. There is still some uncertainty about the adverse effects the vaccine could have; correlating the possible presence of alterations in the oral microcirculation with vaccination would also fill a deficiency of scientific studies of this type not yet present in the literature.

## 2. Materials and Methods

This study took place at the Odontostomatology Department of the University of Palermo between September 2021 and June 2022. We enrolled 18 subjects, aged between 20 and 35 years, healthy and without systemic or topical diseases correlated to alteration of the microcirculation of the oral cavity; all patients received two doses of anti-Covid-19 vaccine: 13 patients were vaccinated with Pfizer and 5 with Moderna.

All patients consented to carry out the videocapillaroscopic examination and to archive and use the data obtained for scientific purposes, in compliance with the regulations on privacy and the processing of personal data.

The study group was selected based on the following criteria:

- absence of pathological conditions that could cause alterations in the peripheral microcirculation of the oral mucosa such as: rheumatoid arthritis, Sjogren’s syndrome, oral lichen planus, pemphigus, pemphigoid, scleroderma, Hashimoto’s thyroiditis, hypertension, diabetes mellitus
- no taking of drugs
- good level of oral hygiene and good oral health [38]
- absence of exposure to risk factors, such as tobacco smoke and alcohol
- absence of exposure to radio and chemo-therapeutic agents
- not having contracted COVID-19 infection
- have had two doses of the anti-COVID-19 vaccine.

All patients of the research underwent an oral and videocapillaroscopic examination conducted by a polarized light video capillaroscope (150× magnification) which, exploiting the endogenous chromophores and therefore the hemoglobin inside the red blood cells, allows to obtain very contrasted images (what is observed is not the capillary but the red blood cells inside it).

This exploits the ability of tissues to reflect polarized light on a given wavelength, absorbed instead by endogenous chromophores in hemoglobin. As a result, the red blood cells appear contrasted in black on the light reflective background.

More specifically, the videocapillaroscope used is based on an integrated hardware–software system known as Horus, which uses a high-sensitivity monochrome sensor; it has 640 × 480 pixels capable of capturing videos with a maximum rate of 120 frames per second. The acquisition was made following a strict protocol that standardized the search facilitating subsequent processing by software [39]. A graphical interface in augmented reality has allowed both the objective measurement of the morphometric characteristics of the capillaries (average elongation, direction, caliber and tortuosity) and the study of the blood flow inside them. The software performs an initial identification of the capillaries by segmenting and separating them from the rest of the image, and then, through a stabilization algorithm, passes to the reconstruction of the capillary, which is carried out starting from several consecutive frames [39]. Once the capillaries were correctly segmented, a pseudo-three-dimensional reconstruction was performed using the same software.

The procedure execution protocol has been standardized for all patients; in fact, the capillaroscopic examination was conducted under standardized temperature and lighting conditions; (room temperature of 24 °C ± 1 °C, neon light-6500 °K). All acquisitions were conducted in the morning with the subjects in a sitting position. A complete capillaroscopic mapping of the oral cavity was made for each subject examined; the mucous membranes investigated were the following: cheek, lower labial, upper labial, gingival and tongue. For each site, a video (25 s) was recorded, which was used for data processing and analysis. All the acquisitions and subsequent processing of the capillaroscopic images were carried out by the same operator. Referring to the classification of capillaroscopic pictures and microangiotectonics of the microcirculation according to Curri, the videocapillaroscopic pictures examined were of type I (arrangement of the capillary loops parallel to the mucosal surface, typical of the microcirculation of the labial and genial mucosa) [40,41]. 

The capillaroscopic data evaluated were of the parametric and non-parametric type.

The parametric data were the following:

1. Capillary density (DC), defined as the number of loops per unit of surface (mm^2^); this parameter, as emerged from previous capillaroscopic studies on the oral mucosa, is the one most directly related to the inflammatory state of the tissues and angiogenetic activity; the general values of capillary density observed in the capillaroscopic studies fluctuated between 12 and 20 capillaries per mm^2^. The study of the literature shows that the data on the capillary density of the microcirculation of the oral cavity deduced by videocapillaroscopy have a perfect correspondence with the histological evaluations in vitro [42,43]. 

2. Loop diameter, (WC) that is the maximum width of the capillary loop, defined as the distance between the tangents to the capillary loop, parallel to the long axis of the loop itself, at the points of maximum convexity. (between 9 and 14 μ.)

3. Capillary loop length (LC), defined as the distance between the beginning of the loop, the junction between the starting point of the afferent head and the end point of the efferent head, and the top of the loop itself; the normal range of the length of a capillary fluctuates between 150 μm and 500 μm (for straight-line capillaries). Average LC = 11 ± 0.8 µ.

4. The caliber or the diameter of the section of a vase, which is different from the diameter of the loop, because the latter is the result of the sum of the two ends of a loop including the interposed space.

Non-parametric data: the visibility of the loops and their position relative to the mucosal surface were evaluated.

The visibility of the capillary loops was evaluated as follows:

Score 1: easy to focus (less than 30 s from the start of the exam)
Score 2: relatively easy to focus (between 30 s and 2 min from the start of the exam)
Score 3: difficult to focus (more than 2 min from the start of the exam)
Score 4: impossible to focus.

The position of the loops with respect to the surface was also evaluated:

- loop parallel to the surface
- loop perpendicular to the surface
- ring is parallel and perpendicular.

In all sites analyzed, the visibility of the microcirculation is equal to 1 and the position of the loops mainly of type A. The results obtained are various images, which can be divided by characteristics (capillary density, loop length, loop diameter, tortuosity) and by type of graph (all the values shown in graphs are in pixels).

The graphs were obtained starting from the evaluations of the mean and standard deviation observed in each single video-frame examined. Then, for each frame, the software identified the vessels and calculated the number of vessels identified, the number of overlaps between vessels, mean and standard deviation (calculated from the single values on the individual vessels), for various characteristics. Each parameter collected was then compared between the two groups of vaccinated patients, precisely to assess whether or not there were significant differences.

The software-assisted analysis on parametric data was performed only on four of the six regions examined in the study. Each numerical value obtained from the software, which is expressed in pixels, has been multiplied by the conversion coefficient in microns of the optics 150×, or by 2.65625. This data was provided by the manufacturer of the video capillaroscope.

The mean capillary density value of the vaccinated patients was obtained considering that the image is a rectangle consisting of 640 × 480 pixels; it follows that (640 pixels × 2.65625 microns per pixel) × (480 pixels × 2.65625 microns per pixel) = 2.1675 square millimeters.

This means that the surface included in an image is 2.1675 mm^2^, so taking the representative value of the number of vessels in a video and dividing it by this number can obtain the density in n/mm^2^ (number of vessels per square millimeter).

Finally, the average of the values obtained for each parameter was calculated and the data obtained were grouped and subjected to statistical analysis to verify the significance of the differences between healthy patients vaccinated with Pfizer and those vaccinated with Moderna.

The test chosen is the T-test; (parametric statistical test to evaluate the means of one or two populations by hypothesis testing).

## 3. Results

- *LEFT CHEEK MUCOSA*

**Density/mm^2^:**

First dose

The mean value (patients vaccinated with Pfizer) is 27.92 µm/mm^2^.
The mean value (patients vaccinated with Moderna) is 21.03 µm/mm^2^.
The difference between these two values is statistically negligible and not significant.

Second dose

The mean value (patients vaccinated with Pfizer) is 29.91 µm/mm^2^.
The mean value (patients vaccinated with Moderna) is 22.42 µm/mm^2^.
The difference between these two values is statistically negligible and not significant.

Capillary loop length:

The mean value (patients vaccinated with Pfizer) is 115.06 µm.
The mean value (patients vaccinated with Moderna) is 116.28 µm.
The difference between these two values is statistically negligible and not significant.

Loop diameter:

The mean value (of patients vaccinated with Pfizer) is 30.59 µm.
The mean value (of patients vaccinated with Moderna) is 31.76 µm.
The difference between these two values is statistically negligible and not significant.

Section gauge:

The mean value (of patients vaccinated with Pfizer) is 7.01 µm.
The mean value (of patients vaccinated with Moderna) is 7.41 µm.
The difference between these two values is statistically negligible and not significant.

- *RIGHTCHEEK MUCOSA*

**The density of the capillaries per mm^2^:**

First dose

The mean value (of patients vaccinated with Pfizer) is 26.69 µm/mm^2^.
The mean value (of patients vaccinated with Moderna) is 16.34 µm/mm^2^.
The difference between these two values is statistically significant.

Second dose

The mean value (of patients vaccinated with Pfizer) is 28.73 µm/mm^2^.
The mean value (of patients vaccinated with Moderna) is 15.70 µm/mm^2^.
The difference between these two values is statistically significant.

Length of the capillary loop:

The mean value (of patients vaccinated with Pfizer) is 127.79 µm.
The mean value (of patients vaccinated with Moderna) is 123.46 µm.
The difference between these two values is statistically negligible and not significant.

Loop diameter:

The mean value (of patients vaccinated with Pfizer) is 29.08 µm.
The mean value (of patients vaccinated with Moderna) is 30.18 µm.
The difference between these two values is statistically negligible and not significant.

Caliber of the vessel section:

The mean value (of patients vaccinated with Pfizer) is 7.49 µm.
The mean value (of patients vaccinated with Moderna) is 8.13 µm.
The difference between these two values is statistically negligible and not significant.

- *INFERIOR LABIAL MUCOSA*

**Density of capillaries:**

First dose

The mean value (of patients vaccinated with Pfizer) is 30.66 µm/mm^2^.
The mean value (of patients vaccinated with Moderna) is 24.08 µm/mm^2^.
The difference between these two values is statistically negligible and not significant.

Second dose

The mean value (of patients vaccinated with Pfizer) is 32.75 µm/mm^2^.
The mean value (of patients vaccinated with Moderna) is 27.79 µm/mm^2^.
The difference between these two values is statistically negligible and not significant.

Length of the capillary loop:

The mean value (of patients vaccinated with Pfizer) is 287.46 µm.
The mean value (of patients vaccinated with Moderna) is 283.98 µm.
The difference between these two values is statistically negligible and not significant.

Loop diameter:

The mean value (of patients vaccinated with Pfizer) is 84.58 µm.
The mean value (of patients vaccinated with Moderna) is 82.21 µm.
The difference between these two values is statistically negligible and not significant.

Caliber of the vessel section:

The mean value (of patients vaccinated with Pfizer) is 17.92 µm.
The mean value (of patients vaccinated with Moderna) is 18.94 µm.
The difference between these two values is statistically negligible and not significant.

- *SUPERIOR LABIAL MUCOSA*

**Density of capillaries:**

First dose

The mean value (of patients vaccinated with Pfizer) is 27.35 µm/mm^2^.
The mean value (of patients vaccinated with Moderna) is 23.33 µm/mm^2^.
The difference between these two values is statistically negligible and not significant.

Second dose

The mean value (of patients vaccinated with Pfizer) is 30.41 µm/mm^2^.
The mean value (of patients vaccinated with Moderna) is 25.97 µm/mm^2^.
The difference between these two values is statistically negligible and not significant.

Length of the capillary loop:

The mean value (of patients vaccinated with Pfizer) is 291.17 µm.
The mean value (of patients vaccinated with Moderna) is 307.62 µm.
The difference between these two values is statistically negligible and not significant.

Loop diameter:

The mean value (of patients vaccinated with Pfizer) is 79.05 µm.
The mean value (of patients vaccinated with Moderna) is 84.91 µm.
The difference between these two values is statistically negligible and not significant.

Caliber of the vessel section:

The mean value (of patients vaccinated with Pfizer) is 18.05 µm.
The mean value (of patients vaccinated with Moderna) is 18.95 µm.
The difference between these two values is statistically negligible and not significant.

A comparison was made between data on capillaries from the oral mucosa of vaccinated patients included in the study and similar data in the literature [44]. 

From data in the literature, and from our experience, it seems that the most significant capillaroscopic parameter of inflammation and the most objectively measurable, repeatable and comparable (even with studies carried out by various researchers) is capillary density. For this reason, the comparison with the literature data was carried out only for these parametric information.

Comparing the inferior capillary density value of the labial mucosa of patients vaccinated with Pfizer and that of patients vaccinated with Moderna, respectively, with the value of the literature, the resulting difference is statistically significant as demonstrated by the T test, since the value of the t-statistic is greater than the critical t.

The mean value of the capillary density of patients vaccinated with Pfizer is 32.75 n/mm^2^ and of patients vaccinated with Moderna is 27.79 n/mm^2^, while the mean capillary density value found in the literature in healthy non-vaccinated patients in the pre-Covid era is 12.80 n/mm^2^.

Comparing the capillary density value of the upper labial mucosa of patients vaccinated with Pfizer and that of patients vaccinated with Moderna, respectively, with the value of the literature, the resulting difference is statistically significant as demonstrated by the T test, since the value of the t-statistic is greater than the critical t.

The mean value of the capillary density of patients vaccinated with Pfizer is 30.41 n/mm^2^ and of patients vaccinated with Moderna is 25.97 n/mm^2^, while the mean capillary density value found in the literature in healthy non-vaccinated patients in the pre-Covid era is 12.80 n/mm^2^.

This study analyzed the samples from the patients after the first dose of vaccine also. All data were comparable to those obtained from the analysis carried out after the second dose. The only statistically significant data with the literature were the same.

An example of the graphics developed by the software can be seen in Figure 2, Figure 3, Figure 4, Figure 5, Figure 6, Figure 7, Figure 8 and Figure 9.

## 4. Discussion

Videocapillaroscopy allows the study of the morphological and architectural structure of the microcirculation and its hemodynamic conditions by a polarized light videomicroscope, whose images are saved and analyzed through dedicated software, which extrapolates the main hemodynamic and morphometric information about capillaries [45,46,47,48,49].

From the scientific literature it is clear that capillaroscopy allows us to delineate that the reactivity of the microvascular-tissue system to exogenous stimuli, especially pharmacological ones, represents one of the most reliable elements for the microvascular diagnosis.

This capillaroscopic study showed an increase in capillary density from the comparison between the mean labial capillary density of vaccinated patients and the reference mean capillary density value of the literature; an increase in capillary density is a sign that can be attributed to an increase in angiogenic activity.

Inside an inflammatory process, the consequent reduction in the tension of the transported oxygen activates the numerous growth factors, including VEGF (vascular endothelial growth factor), which in turn promotes neoangiogenesis; the growth of new capillary loops and the increased density is probably a compensatory mechanism to ensure sufficient perfusion.

The altered vascular circulation of the mucosa is a predisposing factor for all the numerous pathologies of inflammatory etiology: the natural defense mechanisms, the properties of cell-mediated immunity, the complement system, are considerably less effective, also due to the reduced cellularity linked to the reduction of perfusion.

An exalted angiogenesis seems to be at the basis of chronic inflammatory processes, as a consequence of also chronic inflammatory stimuli.

VEGF which is one of the main growth factors of the vascular endothelium, (the proteins that regulate angiogenesis) is a key molecule of angiogenesis. 

VEGF can be considered as a biochemical factor of angioflogosis just as capillary density is a capillaroscopic parameter of inflammation. VEGF and capillary density are two different ways of observing the variation in vascular density and therefore inflammation.

Inside the blood vessels the endothelium is in a quiescent state; however, it preserves the ability to respond to stimuli, direct or indirect, hypoxia, inflammatory or as a result of mechanical stress, and to activate itself due to the release of cytokines and locally produced growth factors to compensate for the lack of oxygen and nutrients. These biochemical mediators, through signaling pathways, can cause hemodynamic modifications of pre-existing vessels and increase the micro-permeability of the endothelial layer (dissolution of cellular junctions and fenestrations). 

VEGF is, both in physiological and pathological conditions, one of the most important proteins involved in the regulation of vascular endothelial activity and in angiogenesis; there are several mechanisms involved in its gene regulation: first of all, the tissue concentration of O_2_ in hypoxic conditions and glucose deficiency.

The immune system plays an important role in regulating angiogenesis, and many studies indicate that leukocytes and proinflammatory cytokines (TNF, IL-1, IL-8, IFN, TGF) are able to induce vascular proliferation.

Observing the results obtained from the comparison of the parametric data of the microcirculation of the oral cavity between the Pfizer vaccine and the Moderna vaccine, no statistically significant differences can be deduced in terms of the morphometric characteristics of the microcirculation of the oral cavity.

Contrary to what has long been believed, the microcirculation does not present a monotonous, repetitive structural organization but, on the contrary, a marked inter-individual and intra-individual variability.

Through capillaroscopy, it has been widely documented how the different cutaneous or mucous regions differ from each other in position, shape, size and micro-angiotectonics (microcirculatory organization) [3,32,42,43,44,45,46,47,48,49].

This peculiar arrangement of the capillaries is strictly dependent not only on the anatomical district (the capillary bed assumes different shapes and characteristics from one organ to another; the capillary density of a given tissue is in fact directly proportional to the metabolic activity of its cells, which involves a greater demand for blood), but it is also related to age, sex, pathologies in progress, environmental factors with chronic exposure [42,43,44,45,46,47,48,49].

## 5. Conclusions

EMA, COVID-19 subcommittee of the WHO Global Advisory Committee on Vaccine Safety (GACVS) and MHRA have reviewed the risk of thrombosis after vaccination with AstraZeneca, again agreeing that the benefits outweigh the risks.

The risks and benefits of current vaccines must be compared with the real possibility of contracting the disease and developing long-term complications based on available clinical evidence and avoiding unjustified bias.

This is the first time that a similar study has been undertaken and it is also the first time that this type of software has been used; excellent potentialities have emerged on this software which over time can also be optimized for an increasingly accurate and precise use in the diagnosis of capillary microcirculation.

## Figures and Tables

**Figure 2:**
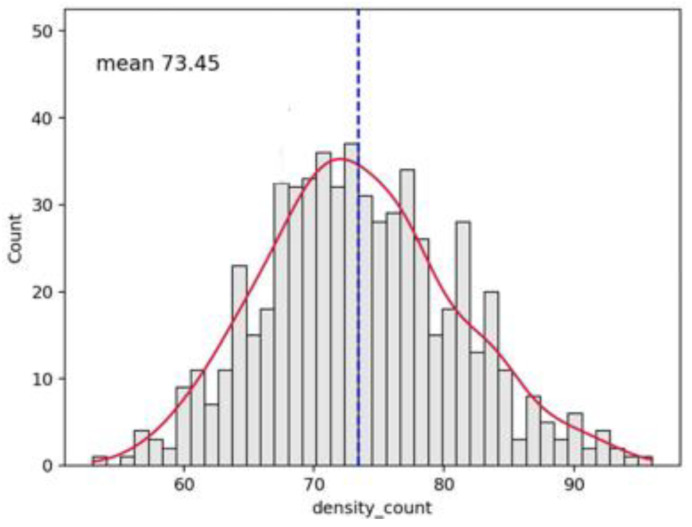
Histogram distribution of capillary density for the whole video (see Figure 1). The dashed line indicates the mean value (73.45 in this case); the red line interpolates the data for clearer reading.

**Figure 3:**
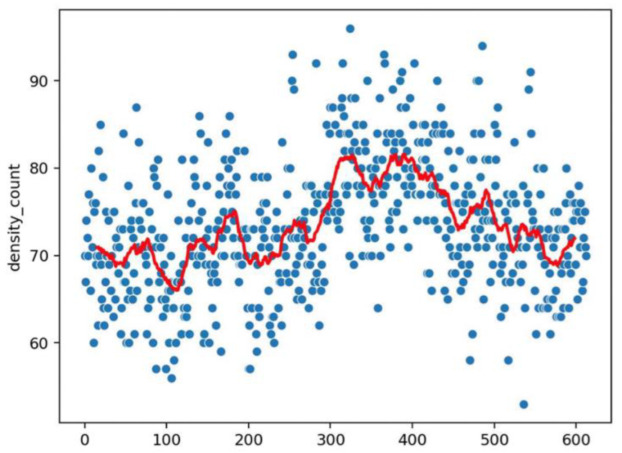
Dot representation of Figure 2. In this case, we can detect eventual anomalies during the acquisition (e.g., out of focus). The *x* axis represents the frame of the video; the *y* axis is the density for the considered frame; the red line interpolates the data for clearer reading.

**Figure 4:**
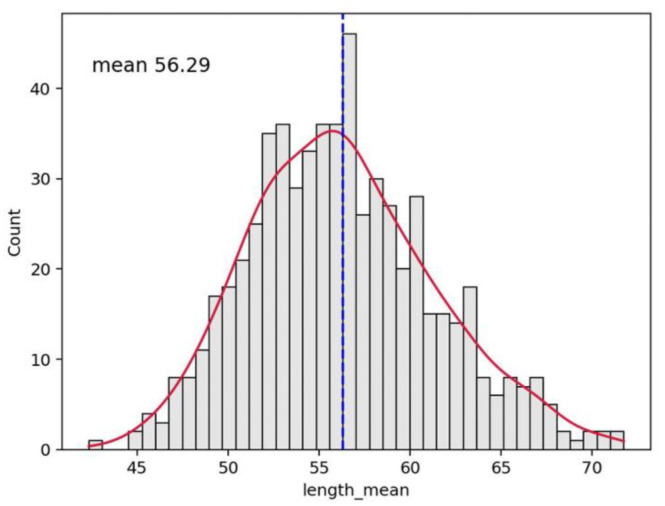
Histogram distribution of loop length for the whole video. As for Figure 2 the dashed line indicates the mean value (56.29 in this case); the red line interpolates the data for clearer reading.

**Figure 5:**
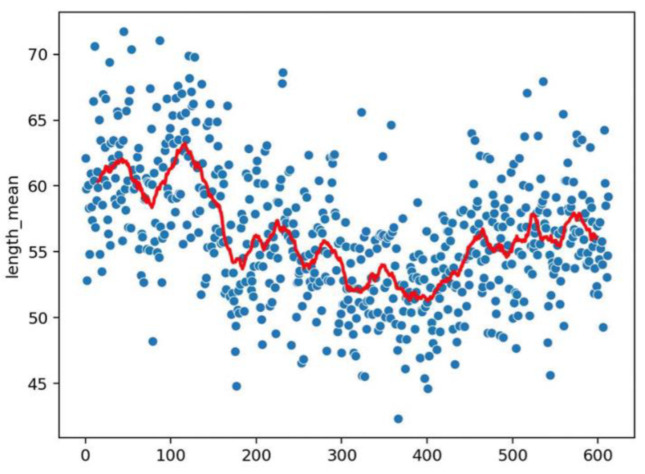
Dot representation of Figure 4. As for Figure 3 we can detect eventual anomalies during the acquisition (e.g., out of focus).

**Figure 6:**
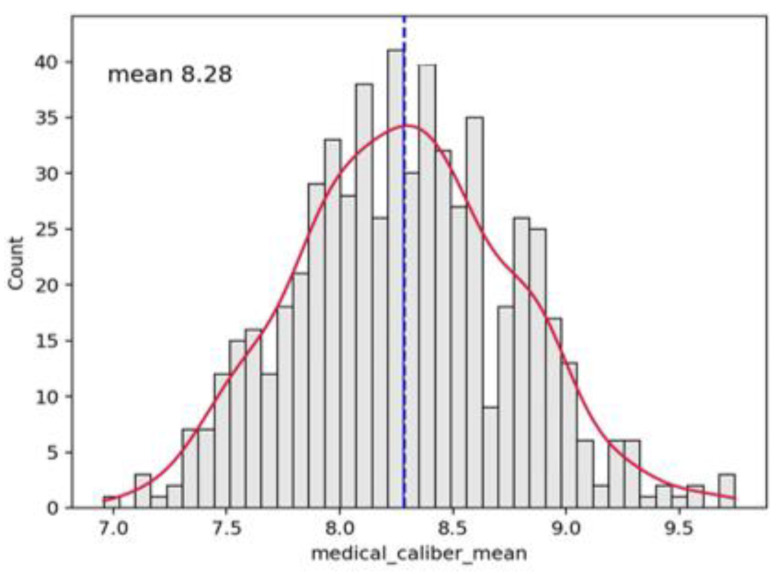
Histogram distribution of loop diameter for the whole video. The dashed line indicates the mean value equal to 8.28 in this case.

**Figure 7:**
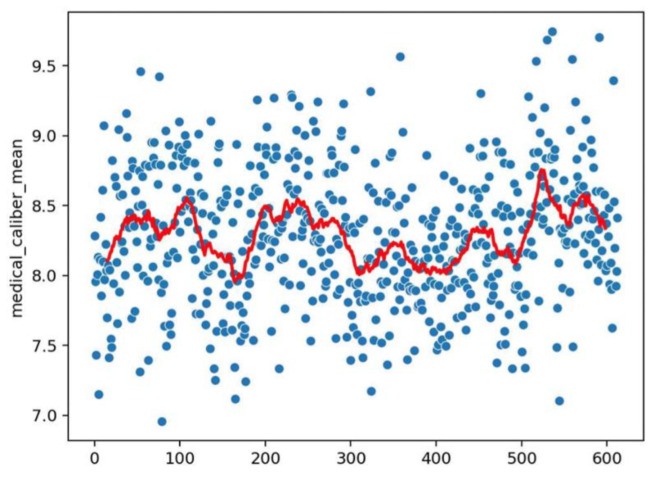
Dot representation as for Figure 3 but with respect to the information of Figure 6.

**Figure 8:**
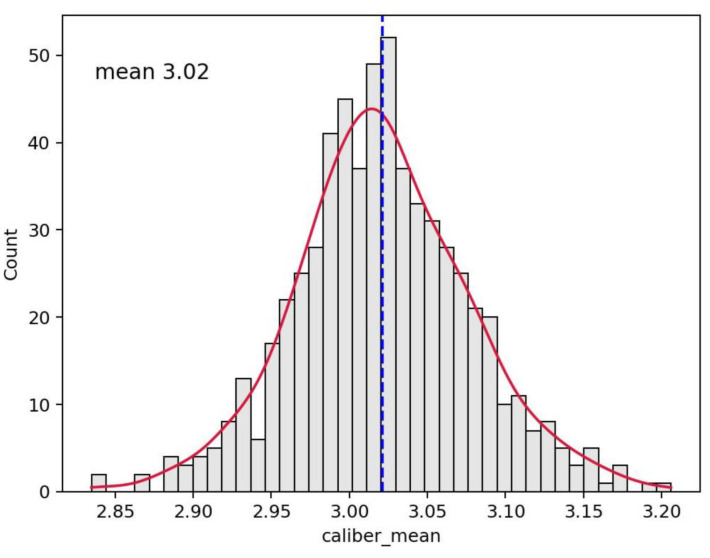
Histogram distribution of section gauge for the whole video. The dashed line indicates the mean value equal to 3.02 in this case.

**Figure 9:**
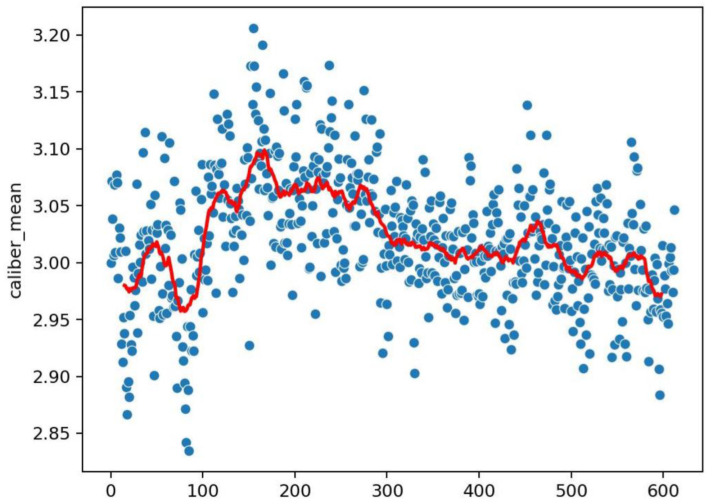
Dot representation as for Figure 3 but with respect to the information of Figure 8.

## Data Availability

Not applicable.
